# Modest advances, persistent inequalities: child mortality in Brazil from 2010 to 2022

**DOI:** 10.11606/s1518-8787.2025059006452

**Published:** 2025-07-07

**Authors:** Antonio Fernando Boing, Alexandra Crispim Boing

**Affiliations:** I Universidade Federal de Santa Catarina Programa de Pós-graduação em Saúde Coletiva Florianópolis SC Brasil Universidade Federal de Santa Catarina. Programa de Pós-graduação em Saúde Coletiva. Florianópolis, SC, Brasil

**Keywords:** Infant Mortality, Child Mortality, Socioeconomic Disparities in Health, Brazil

## Abstract

**OBJECTIVE:**

To analyze the evolution of socioeconomic and regional inequalities in mortality among children under five years of age in Brazil between 2010 and 2022.

**METHODS:**

Data from 37,639,196 live births (LB) and 563,711 deaths among children under five years during the period were analyzed. Mortality rates for infants (<one year-old, IMR) and children aged one to four years were calculated. For inequality analysis, municipalities were grouped according to deciles of the 2010 Municipal Human Development Index (M-HDI). The Slope Index of Inequality (SII) and the Relative Index of Inequality (RII) were calculated. The excess mortality was estimated by applying the mortality rates observed in the highest M-HDI decile to the other deciles. The spatial distribution of deaths was analyzed according to the country’s microregions.

**RESULTS:**

The IMR decreased from 13.0 to 12.7 per thousand LB between 2010 and 2022, while mortality among children aged one to four years remained stable at 2.5 per thousand LB in 2010-2022. There was only a slight reduction in inequalities, with the SII oscillating from -5.63 to -4.91 in IMR and from -2.42 to -1.71 in mortality among children aged one to four years between 2010-2022. In 2022, municipalities with lower M-HDI had mortality rates 49.0% and 93.0% higher than those with higher M-HDI in IMR and mortality among children aged one to four years, respectively. Inequalities in IMR were more pronounced in nutritional, metabolic, and endocrine diseases, where mortality was four times higher in municipalities with lower M-HDI. There was an excess of 76,832 child deaths in Brazil between 2010-2022. In 2022, 42.2% of the microregions in the North were among the top 100 with the highest IMR, compared to only 3.2% in the South.

**CONCLUSIONS:**

There was a slight reduction in child mortality, but significant socioeconomic and regional inequalities persisted in Brazil.

## INTRODUCTION

Globally, the infant and child mortality rates fell from 64 and 93 deaths per thousand live births in 1990 to 28 and 37 in 2022 respectively, reflecting significant progress in child health^1^. However, these improvements have not occurred equitably between countries and population groups. The average infant and child mortality rates in low-income countries were approximately 12 times higher in 2021 than those observed in high-income countries^2^. These variations highlight the disparities in access to better general living conditions, health care, nutrition, basic sanitation, and other socioeconomic conditions.

Brazil has achieved a notable reduction in infant mortality since the 1980s. In 2022, the observed value was approximately 48.0% lower than in 2000 and 71.0% lower than in 1990^3^. Even so, this 2022 figure is almost four times higher than the average for high-income countries^2^. The targets of the Sustainable Development Goals call for countries to have a mortality rate among children under five less than 25 deaths per 1,000 live births by 2030, a limit that Brazil has reached since 2004^3^. However, the goal also calls for an end to preventable deaths in this age group and, in 2022, Brazil had 24,349 preventable deaths among children under five^4^.

In addition to the decrease in infant mortality, regional inequalities were also reduced, especially in the 1990s and the first decade of the 21st century^5^. Different social policies and achievements have contributed to these advances. Improvements in material living conditions, increased family income, and schooling are cited as important factors^6^. Moreover, improving the supply of health services, expanding the coverage of the Family Health Strategy and direct income transfers are successful policies with evidence of a positive impact on reducing infant deaths^7^.

However, the second half of the 2010s in Brazil was marked by an economic crisis, reforms in social and economic policies, and limited public spending, which particularly affected health and social programs and actions with the potential to influence infant mortality. Previous studies have estimated the potential impact of these changes on health, indicating significant negative effects on population indicators^8^. The need to assess whether the downward trends in inequalities would be reversed has also been highlighted^9^. The Covid-19 pandemic has heightened concern about children’s indicators, particularly given the uneven way in which it has affected Brazil and the high burden of the disease in the country^10^.

The aim of this study was to analyze the evolution of socioeconomic and regional inequalities in under-five mortality in Brazil between 2010 and 2022, identifying patterns and groups of diseases with greater inequality and estimating excess deaths in municipalities with the lowest Human Development Index (HDI).

## METHODS

Data on births and deaths of all children under five years of age living in Brazil between 2010 and 2022 were analyzed by: age group (under one year of age and between one and four years of age), underlying cause of death (according to the chapters of the tenth revision of the International Classification of Diseases - ICD-10), and municipality and micro-region of residence.

The registration of deaths in Brazil is compulsory and the data is recorded on a death certificate which is used for the civil registration of the event in official registry offices. All death certificates are collected by municipal health departments and their data entered into the national Mortality Information System (SIM), managed by the Ministry of Health. Similarly, birth registrations in Brazil are also compulsory and make up the Live Birth Information System (Sinasc), which aggregates data on all births in the country.

Although SIM and Sinasc coverage has increased significantly since the 2000s, under-registration of vital statistics still persists in Brazil. For this reason, the Brazilian Institute of Geography and Statistics (IBGE) calculates the rates of under-registration and under-reporting of births and deaths in the country and makes them available openly^11^. This data were used in this study, incorporating the birth estimates provided by the IBGE for each municipality and the underreporting estimates associated with the SIM data, with the aim of correcting the numbers of deaths of children under five. Since 2015, the IBGE has provided corrected vital statistics based on the capture-recapture technique. Thus, for the period from 2010 to 2014, the corrections observed in 2015 and 2016 were applied for each municipality, considering the proximity in time and the need to ensure the consistency of the adjustments with the most recent data available and following the same method. The global correction values were applied to each cause of death chapter. In addition, a sensitivity analysis was carried out, replicating all the calculations from the raw SIM and SINASC data.

The 5,565 Brazilian municipalities emancipated in 2010 and therefore with census data available for that year were aggregated according to the Municipal Human Development Index (M-HDI) deciles, to compose groups with a number of deaths and live births that allowed for stability in the indicators. The M-HDI is an adaptation of the United Nations (UN) HDI applied to measure human development at the municipal level in Brazil. We used the index calculated for 2010, the initial year of the analysis. This data is available in the Atlas of Human Development^12^.

First, the mortality rates in each of the M-HDI deciles were calculated for each year analyzed, allowing for a graphical evaluation of the historical series in each group. Then, for the final year of the historical series, 2022, the mortality rate in each M-HDI decile was analyzed according to the 10 ICD-10 chapters with the highest occurrence of deaths in each age group. The volume of deaths in the other chapters was small, limiting the possibility of segmented analyses. The chapters with calculated rates were: (1) Some infectious and parasitic diseases, (2) Neoplasms, (3) Diseases of the blood and hematopoietic organs, (4) Endocrine, nutritional, and metabolic diseases, (6) Diseases of the nervous system, (9) Diseases of the circulatory system, (10) Diseases of the respiratory system, (11) Diseases of the digestive system, (16) Some conditions originating in the perinatal period, (17) Congenital malformations, deformities, and chromosomal anomalies, (18) Symptoms, signs, and abnormal clinical and laboratory findings, (20) External causes. In addition, the mortality ratios for each chapter were calculated between the extreme HDI-M deciles.

To analyze inequalities in the occurrence *of* deaths, the Slope Index of Inequality (SII) and the Relative Index of Inequality (RII) were estimated for each year. The SII is considered a complex measure of inequality that estimates the differences in mortality rates between the extremes of socioeconomic distribution, considering the composition of the entire sample. The RII is a relative measure of inequality and also considers both the distribution of the population between different groups and the relative position of each group.

Next, the excess deaths were calculated for each year and M-HDI decile, assuming for all deciles the annual mortality values observed in the group with the highest M-HDI. The excess deaths were calculated using the following formula considering age (i) and year (j):


$$\sum \left[{0\mathrm{btObs}}_{n}(i,j)-\left({\mathrm{txd}}_{10}(i,j)*{\mathrm{nv}}_{n}(i,j)\right)\right]$$

where <0btObs_n_(i,j)> is the number of deaths in decile n at age i and year j; < txd_10_(i,j)> is the mortality rate at age i and year j in the decile with the highest M-HDI; and < nv_n_(i,j)> is the number of live births at age i and year j in decile n.

Finally, to explore the occurrence of regional inequalities in the Brazilian territory, mortality rates were calculated according to the micro-regions of residence. The IBGE divides Brazil into 558 micro-regions according to geographical proximity, socioeconomic characteristics, and functional integration. Geographical aggregation allowed for more stable calculations compared to analysis by municipality, given the existence in Brazil of many municipalities with small resident populations. The maps and geographical coordinates were obtained from the IBGE, and the maps were made in Python 3.4. The other analyses were carried out in Stata 15. All mortality and birth data are anonymized and in the public domain, and is made available by the Ministry of Health^13^. This means that the study did not need to be examined by a research ethics committee.

## RESULTS

We analyzed data on 37,639,196 live births, 483,900 deaths among children under one year old, and 79,811 deaths among children aged one to four. The number of live births was 2.91 million in 2010, peaking in 2015 (3.08 million), but decreasing in the following years until it reached 2.57 million in 2022. The infant mortality rate decreased from 13.0 per 1,000 live births to 12.7 per 1,000 live births between 2010 and 2022 (Table). However, this decrease was only sustained in the first five-year period analyzed. The lowest value in the series was observed in 2020, but it increased in the following years, reaching the same average value in 2022 as in the four-year period from 2014 to 2017. In the case of mortality among children aged one to four, after falling until 2020, the rate rose again in 2021 and 2022 (Table). At the end of the period, after 12 years, the nominal value was identical to that observed in 2010.

**Table:** Deaths, mortality rate, Slope Index of Inequality (SII), Relative Index of Inequality (RII), and ratio and absolute difference between extreme deciles of the infant mortality rate and the mortality rate among children aged one to four according to the extreme deciles of the Municipal Human Development Index. Brazil, 2010 to 2022.

Year	Infant mortality					
	Deaths	Rate	SII (95%CI)	RII (95%CI)	D1/D10	D1/D10
2010	40,947	13.0	-5.63 (-6.60 to -4.60)	0.69 (0.64 to 0.74)	1.48	5.90
2011	40,783	13.7	-5.61 (-6.78 to -4.43)	0.69 (0.63 to 0.74)	1.48	5.65
2012	40,226	13.6	-6.16 (-7.19 to -5.12)	0.66 (0.61 to 0.71)	1.55	6.42
2013	40,055	13.6	-6.68 (-7.45 to -5.91)	0.64 (0.60 to 0.67)	1.56	6.57
2014	39,503	13.0	-5.77 (-7.29 to -4.25)	0.66 (0.59 to 0.73)	1.56	6.39
2015	38,662	12.6	-5.14 (-6.89 to -3.39)	0.68 (0.60 to 0.77)	1.54	5.93
2016	37,240	12.9	-6.24 (-7.42 to -5.06)	0.64 (0.58 to 0.69)	1.58	6.46
2017	36,932	12.5	-5.64 (-7.01 to -4.26)	0.66 (0.59 to 0.72)	1.53	5.93
2018	36,562	12.3	-4.74 (-5.77 to -3.72)	0.70 (0.64 to 0.75)	1.47	5.06
2019	35,943	12.5	-4.98 (-6.79 to -3.17)	0.69 (0.60 to 0.78)	1.54	5.89
2020	32,046	11.6	-5.07 (-6.00 to -4.14)	0.67 (0.61 to 0.72)	1.50	5.12
2021	32,305	12.0	-5.05 (-7.73 to -2.38)	0.67 (0.54 to 0.81)	1.59	6.28
2022	32,695	12.7	-4.91 (-6.30 to -3.52)	0.70 (0.62 to 0.77)	1.49	5.44
	**Mortality among children aged one to four**					
2010	7,242	2.5	-2.42 (-3.24 to -1.60)	0.41 (0.29 to 0.54)	2.37	2.61
2011	6,877	2.3	-1.82 (-2.28 to -1.37)	0.49 (0.40 to 0.59)	2.07	1.92
2012	6,526	2.2	-1.77 (-2.53 to -1.01)	0.48 (0.34 to 0.63)	2.18	2.07
2013	6,537	2.2	-2.13 (-2.83 to -1.42)	0.42 (0.30 to 0.54)	2.35	2.29
2014	6,302	2.1	-1.80 (-2.87 to -0.74)	0.45 (0.25 to 0.66)	2.37	2.27
2015	5,783	1.0	-1.78 (-2.41 to -1.16)	0.42 (0.29 to 0.56)	2.29	1.90
2016	6,388	2.2	-1.93 (-3.10 to -0.77)	0.45 (0.24 to 0.66)	2.33	2.35
2017	6,056	2.0	-1.74 (-3.01 to -0.47)	0.46 (0.21 to 0.71)	2.30	2.18
2018	5,999	2.0	-1.38 (-1.98 to -0.79)	0.54 (0.39 to 0.68)	1.98	1.62
2019	5,934	2.1	-1.35 (-2.17 to -0.52)	0.55 (0.35 to 0.74)	1.97	1.70
2020	4,683	1.7	-1.48 (-2.07 to -0.90)	0.46 (0.32 to 0.60)	2.30	1.70
2021	5,104	1.9	-1.34 (-2.30 to -0.39)	0.52 (0.28 to 0.75)	2.10	1.74
2022	6,379	2.5	-1.71 (-2.59 to -0.82)	0.53 (0.36 to 0.70)	1.93	1.95

95%CI: 95% confidence interval.

**Figure 1:**
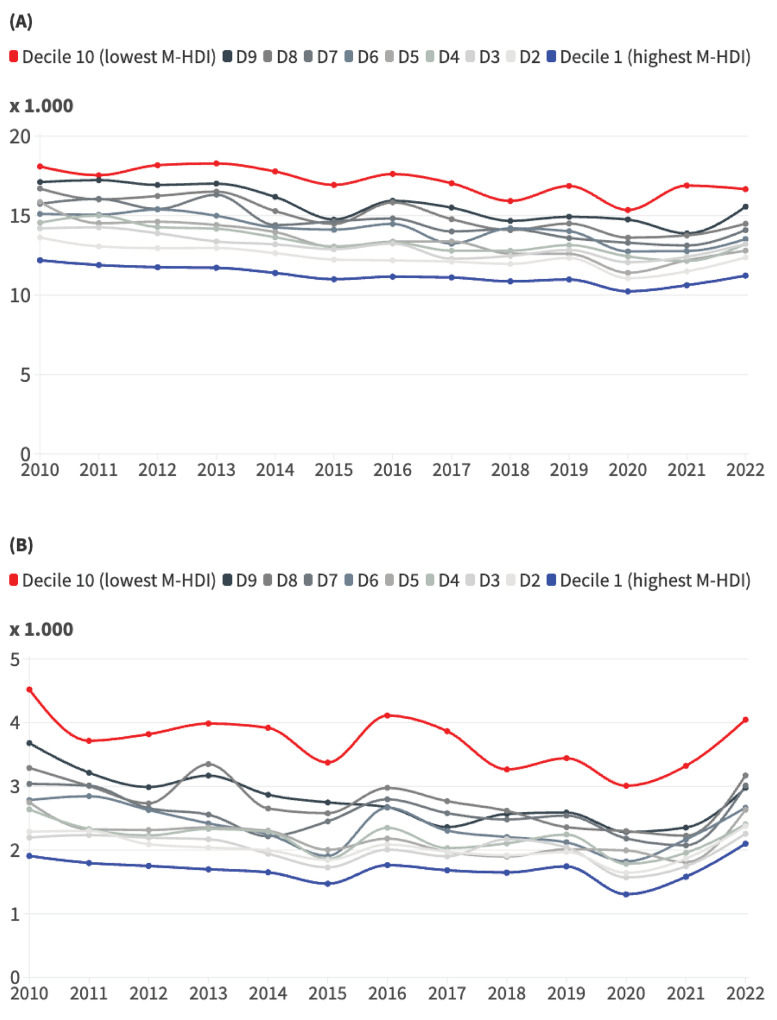
Historical series of infant mortality (A) and mortality among children aged one to four (B) in Brazilian municipalities according to Municipal Human Development Index (M-HDI) deciles. Brazil, 2010 to 2022.

Figures 1A and 1B show that there was a clear gradient of higher mortality rates in the decisions with the lowest M-HDI. In all the years from 2010 to 2022, higher rates were observed in the municipalities with the lowest M-HDI, and lower rates were identified in the municipalities with the highest M-HDI. There was little fluctuation in the positions of municipalities with intermediate indices. The SII fell from -5.63 to -4.91 in infant mortality and from -2.42 to -1.71 in the mortality of children between one and four years of age (Table). The RII for infant mortality, on the other hand, fluctuated slightly downwards in the first four years of the series and then started to rise, being slightly higher than the initial value in 2022. In mortality between the ages of one and four, the RII values increased from 0.41 to 0.53 (Table). Analyzing the ratio between the two extreme deciles, infant mortality was 48.0% and 49.0% higher in the lowest M-HDI decile in 2010 and 2022, respectively. Mortality among children aged one to four was 137.0% and 93.0% higher in the two years, respectively.

**Figure 2:**
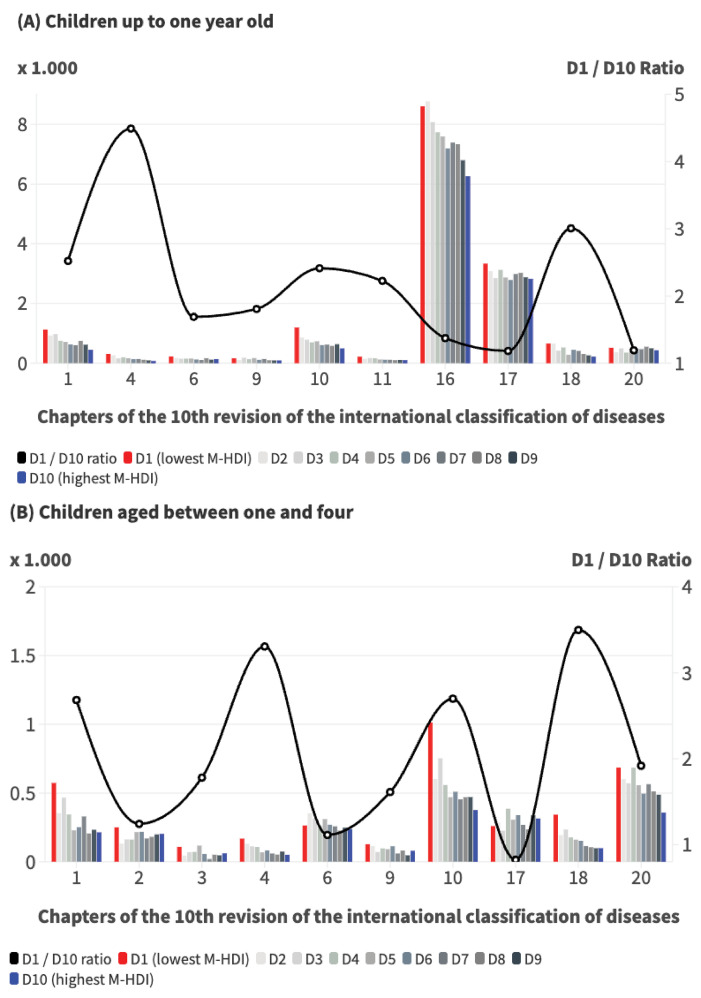
Infant mortality rate (A) and mortality rate among children aged one to four (B) according to chapters of the tenth revision of the International Classification of Diseases according to deciles of the Municipal Human Development Index (M-HDI), and ratio between the deciles with the lowest and highest M-HDI. Brazil, 2022.

These differences varied according to the cause of death. Grouping them according to ICD-10 chapters showed that the infant mortality rate from nutritional, metabolic and endocrine diseases was 4.5 times higher in the lowest HDI-M decile compared to the highest human development decile (Figure 2). The figures were also 3.0 times higher in chapter 18, which includes ill-defined and unknown causes of mortality, and 2.5 times higher in the group of infectious and parasitic diseases. Among children aged one to four, the biggest differences were in the group that includes ill-defined and unknown causes (3.5 times), endocrine, nutritional, and metabolic diseases (3.3 times), respiratory diseases (2.7 times), and infectious and parasitic diseases (2.7 times).

**Figure 3:**
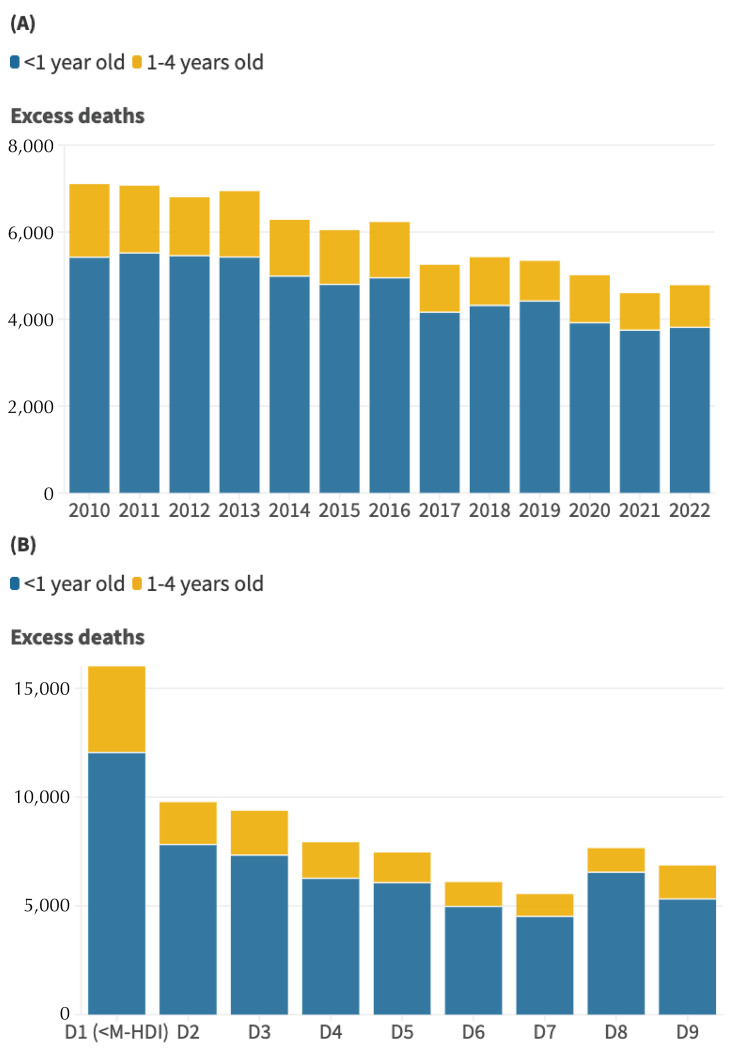
Number of excess deaths comparing infant mortality rates and mortality rates among children aged one to four in the deciles with the lowest Municipal Human Development Index (M-HDI) compared to the rates observed in the decile with the highest M-HDI according to year of death (A) and according to socioeconomic decile for the entire period (B). Brazil, 2010 to 2022.

These inequalities have resulted in a significant number of excess child deaths in the country. If the mortality rates observed in the group with the highest M-HDI were applied to the other deciles, 76,832 deaths of children under the age of five would have been avoided in Brazil between 2010 and 2022 (Figure 3A). Excess child deaths were highest between 2010 and 2013, decreasing especially from 2017 onwards (in line with the reduction in live births), but always remaining above 4,500 per year. The excess death figures were also higher in the lowest M-HDI decile, reaching almost 16,164 excess deaths in these municipalities alone between 2010 and 2022 (Figure 3B).

**Figure 4:**
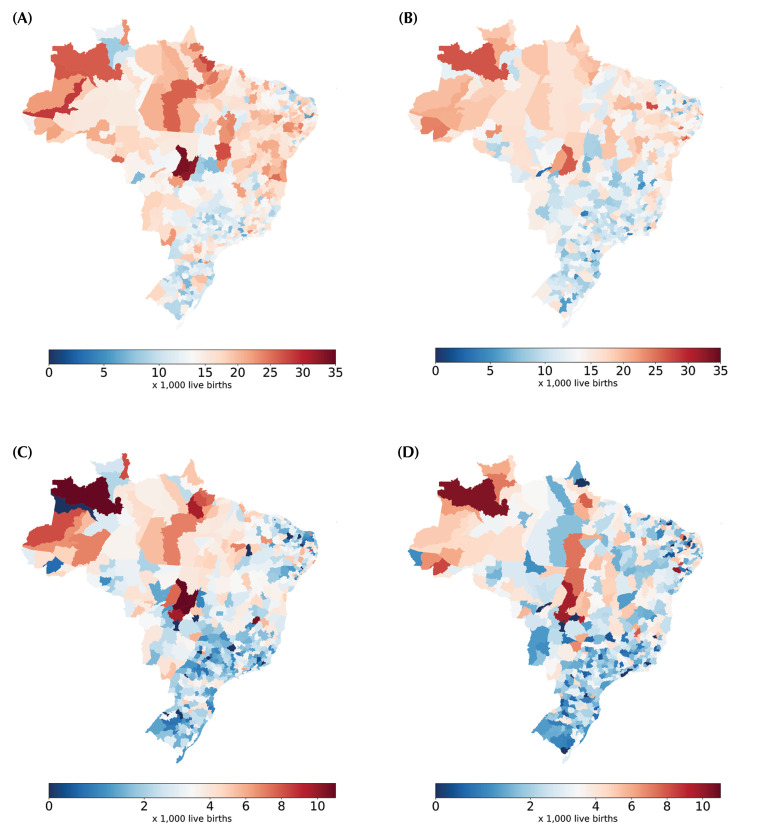
Infant mortality rate in 2010 (A) and 2022 (B) and among children aged one to four in 2010 (C) and 2022 (D) according to the child’s micro-region of residence. Brazil, 2010 and 2022.

The spatial analysis of the two outcomes analyzed also showed the existence of deep and constant regional inequalities in Brazil. It was observed that the North and Northeast regions concentrated most of the micro-regions with the highest mortality rates (Figure 4). In 2022, 42.2% and 28.2% of the micro-regions in the North and Northeast were among the 100 with the highest infant mortality rates, compared to only 5.0%, 3.2% and 17.3% in the Southeast, South, and Central-West, respectively. In addition, only 7.8% and 9.6% of the micro-regions in the North and Northeast were among the 100 with the lowest infant mortality rates, while in the Central-West, South and Southeast the figures were 17.3%, 31.9%, and 23.8%, respectively

In the sensitivity analysis, there were variations in all the measures calculated when using uncorrected SIM and Sinasc data.

However, the differences were of low magnitude and did not change the direction of the associations, as can be seen in the supplementary material^a^.

## DISCUSSION

This study found five important results. Firstly, there was a slight reduction in under-five mortality over the period analyzed, with a small fluctuation between 2016 and 2019 and a worsening in 2022. Secondly, inequalities in mortality persisted throughout the period, with only a slight reduction or stabilization. Thirdly, inequalities were more acute in deaths from nutritional, metabolic, and endocrine diseases, infectious and parasitic diseases, respiratory diseases, and the group of ill-defined causes. Fourthly, if municipalities had the same mortality rates as those observed in the decile with the highest M-HDI, almost 77,000 under-five deaths could have been avoided in Brazil between 2010 and 2022. Finally, there have been profound regional inequalities in mortality over the years, with the worst indicators in the North and Northeast regions and with little fluctuation.

The reduction in mortality observed at some points in the time series reflects improvements in living conditions and access to health services. Previous studies have shown that economic, health and social improvements associated with interventions in primary health care (PHC) and income transfer programs have contributed significantly to the reduction of under-five mortality in Brazil^7,14^. However, the relative stability between 2016 and 2019 and the increase in 2022 indicate that these advances are not definitive achievements. The revision of the National Primary Care Policy (PNAB) in 2017 was cited as a threat to the strengthening of primary care in Brazil by relativizing universal coverage^15^. Freire et al.^16^ observed, in the two years following the approval of the new PNAB, the growth of municipalities that reduced the number of community health agents working in the Unified Health System (SUS). Other measures, such as discouraging multidisciplinary PHC support teams and changes to primary care financing and evaluation, have also been criticized in scientific circles^17^.

At the root of this challenging scenario for health was Constitutional Amendment 95, which established a freeze on public investment in Brazil for 20 years from 2017^18^. The proposal was replaced from 2023; however, it restricted public health funding during an important period of economic crisis^18^. Previous studies have shown significant impacts of measures to de-fund actions in the health sector^8,19^. In addition, compared to 2013, federal funding in 2019 was lower in the areas of housing, education, sanitation, labor, urbanism, rural development, and culture^20^.

Greater inequality in nutritional, metabolic, and endocrine diseases, as well as infectious, parasitic, and respiratory diseases, can be explained by the different exposures that residents of municipalities with different human development indices have. More underprivileged regions in Brazil have greater food and nutritional insecurity^21^, less access to infrastructure, adequate housing and basic sanitation^22^ and greater difficulties in accessing health services^23^. On the other hand, the higher mortality rate from the causes listed in Chapter 18 (Symptoms, signs, and abnormal clinical and laboratory findings) in municipalities with a lower M-HDI may be related to greater limitations in access to accurate diagnoses and adequate health resources, as well as weaknesses in death surveillance and investigation systems.

These structural contexts have the potential to have a more severe impact on vulnerable populations and localities with fewer resources and structured public services. Despite some improvements, inequalities remain significant and persistent. A nationwide study^24^has already shown that between 2013 and 2019 there was an increase in the prevalence of eight chronic diseases and that the inequalities present in their distribution did not change during the period. The study by Sousa et al.^25^ identified that in the first years of the Brazilian economic and political crisis (2015 to 2017) there was a deterioration in the sense of well-being in the country, with a more pronounced reduction among people with less schooling and less social support.

The increases in mortality rates observed in this study in 2021 and 2022 may be at least partially associated with the effects of the Covid-19 pandemic. Shapira et al.^26^ reported a 6.8% increase in the total number of expected infant deaths in 2020 in 83 countries, which would be associated with negative income shocks due to the pandemic. Ahmed et al.^27^argued that the decline in the use of essential health services may explain the increase in infant deaths in the first two years of the pandemic. In 2020, Brazil still showed a drop in under-five mortality. It is possible that, in the first year of the pandemic, mitigation measures contributed to reducing exposure to disease-causing pathogens in pediatric age. In the following years, childhood mortality may have been impacted by the plethora of health services and delayed access to them, by the resumption of social activities in the context of low collective and governmental organization to deal with the pandemic and by the effects of the economic crisis.

Another important finding is that the number of excess deaths was quite significant. These figures reveal the profound inequality that still exists in the country, and which permeates various dimensions of life. Despite the fact that all Brazilians are legally protected by the same fundamental rights and that there is a public and universal health system, there is significant over-mortality in the municipalities with the lowest M-HDI. This shows that the knowledge produced and the technologies available in the country are not made available and accessed with equity. In addition, after more than a decade with little progress in tackling inequalities, it is necessary to assess whether public management is monitoring inequalities and implementing equitable actions based on the best evidence and according to real-time data. A similar pattern was observed in England, with 9,294 excess underage deaths between 2009 and 2020^28^. However, when analyzing excess deaths at all ages, unlike that observed among children in Brazil, the number England increased over time, especially from 2015 and peaking in 2020. When interpreting the Brazilian case, the significant reduction in the number of live births from 2019 onwards stands out.

Spatial inequalities suggest that there are insufficient public policies aimed at regional equity. The concentration of micro-regions with high infant mortality rates in the North and Northeast express challenges such as difficulty in accessing quality health services, poorer infrastructure in the health sector and poorer socioeconomic conditions^19,29-30^. These findings indicate the need for effective public policies focused on reducing regional disparities.

Limitations of this study include the possible residual underreporting of deaths and the variation in data quality between different regions. By using data correction, an attempt was made to address this limitation, but variations in quality may remain, albeit to a lesser extent. In addition, the sensitivity analysis with raw data showed only modest adjustments to the calculated measures. A second limitation is the aggregation of the analyses into M-HDI deciles and micro-regions, which would not capture the heterogeneity that can exist within these groups. However, this measure is important to provide stability to calculated indicators. A third aspect to consider is that changes in policies and the economy over the study period can have complex, long-term effects on infant mortality that have not been fully captured. Finally, aggregate indices, such as the M-HDI, may not adequately capture the complexities of populations with high racial/ethnic diversity, such as Brazil. However, the index reflects socio-economic conditions that are widely known to have a robust impact on child health and offers a uniform instrument that facilitates comparison between different municipalities.

The results have important implications for public policies. Interventions focused on reducing regional and socioeconomic inequalities are essential for improving child health in Brazil. Economic, social and health policies need to be implemented to increase the speed of improvement in general living conditions and the living environment and qualify access to health services. In addition, the Covid-19 pandemic has brought new challenges, accentuating pre-existing inequalities and highlighting the need for resilience in health systems. In addition, there needs to be a broad discussion among managers at all levels about how to monitor and reduce health inequalities.

## Footnotes

a Availabe from the Institutional Repository of the Universidade Federal de Santa Catarina at: https://repositorio.ufsc.br/handle/123456789/260749#:~:text=material_suplementar.pdf.
