# Supplementary material for: Modest advances, persistent inequalities: child mortality in Brazil from 2010 to 2022
Source: Rev Saude Publica. 2025 Jul 7;59:e18. doi: 10.11606/s1518-8787.2025059006452 (PMC12306659; doi:10.11606/s1518-8787.2025059006452)
Supplement: Translation

# Translation: Modestos avanços, persistentes desigualdades: mortalidade de crianças no Brasil de 2010 a 2022

**Authors:** Antonio Fernando Boing, Alexandra Crispim Boing

**Affiliations:** I Brasil Universidade Federal de Santa Catarina. Programa de Pós-graduação em Saúde Coletiva. Florianópolis, SC, Brasil

**Keywords:** Mortalidade Infantil, Mortalidade da Criança, Disparidades Socioeconômicas em Saúde, Brasil

## Abstract

**OBJETIVO:**

Analisar a evolução das desigualdades socioeconômicas e regionais na mortalidade entre menores de cinco anos de idade no Brasil entre 2010 e 2022.

**MÉTODOS:**

Foram analisados dados de 37.639.196 nascidos vivos e 563.711 óbitos ocorridos entre crianças menores de cinco anos no período de 2010 a 2022. Calcularam-se taxas de mortalidade infantil entre menores de um ano de idade (TMI) e de mortalidade entre crianças com um a quatro anos. Para análise das desigualdades, os municípios foram agrupados segundo decis do Índice de Desenvolvimento Humano Municipal (IDH-M) de 2010 e foram calculados o *Slope Index of Inequality* (SII) e o *Relative Index of Inequality* (RII). O excesso de óbitos foi estimado aplicando-se taxas de mortalidade observadas no decil de maior IDH-M aos demais. Realizou-se distribuição espacial dos óbitos segundo microrregiões brasileiras

**RESULTADOS:**

A TMI diminuiu de 13,0 para 12,7 por mil nascidos vivos entre 2010 e 2022, enquanto a mortalidade entre crianças de um a quatro anos foi de 2,5 por mil nascidos vivos nos anos extremos analisados. Houve apenas discreta redução nas desigualdades, com o SII oscilando entre 2010 e 2022 de -5,63 a -4,91 na TMI e -2,42 a -1,71 na mortalidade entre crianças de um a quatro anos. Em 2022, municípios com menor IDH-M apresentaram taxas 49,0% e 93,0% maiores em relação àqueles com maior IDH-M na TMI e na mortalidade entre um e quatro anos, respectivamente. Na TMI, as desigualdades foram mais acentuadas em doenças nutricionais-metabólicas-endócrinas, com a mortalidade sendo quatro vezes maior nos municípios com menor IDH-M. Houve excesso de 76.832 óbitos infantis no Brasil entre 2010 e 2022. Em 2022, 42,2% das microrregiões do Norte estavam entre as 100 com maiores taxas de mortalidade infantil, valor que chegou a apenas 3,2% no Sul.

**CONCLUSÕES:**

Houve pequena redução na mortalidade entre crianças e persistiram significativas desigualdades socioeconômicas e regionais no Brasil.

## INTRODUÇÃO

Globalmente, as taxas de mortalidade infantil e na infância caíram, respectivamente, de 64 e 93 mortes por mil nascidos vivos em 1990 para 28 e 37 em 2022, refletindo progressos significativos na saúde infantil^1^. No entanto, essas melhorias não têm ocorrido de maneira equitativa entre países e grupos populacionais. Os valores médios das taxas de mortalidade na infância e infantil em países de baixa renda foram, em 2021, aproximadamente 12 vezes maiores que os observados nos países de alta renda^2^. Essas variações destacam as disparidades no acesso a melhores condições gerais de vida, de cuidados de saúde, nutrição, saneamento básico e outras condições socioeconômicas.

O Brasil conquistou notável redução da mortalidade na infância desde as décadas de 1980. Em 2022, o valor observado foi aproximadamente 48,0% inferior ao do ano 2000 e 71,0% menor que o de 1990^3^. Ainda assim, esse valor de 2022 é quase quatro vezes maior que a média dos países de renda alta^2^. As metas dos Objetivos de Desenvolvimento Sustentável preveem que os países apresentem em 2030 taxa de mortalidade entre menores de cinco anos de idade inferior a 25 óbitos a cada mil nascidos vivos, limite que o Brasil atinge desde 2004^3^. No entanto, a meta também prevê acabar com as mortes evitáveis nessa faixa etária e, em 2022, o Brasil apresentou 24.349 óbitos evitáveis entre menores de cinco anos^4^.

Adicionalmente à queda da mortalidade infantil, desigualdades regionais também foram reduzidas, sobretudo nos anos 1990 e na primeira década do século XXI^5^. Diferentes políticas e conquistas sociais têm contribuído para esses avanços. Melhoria nas condições materiais de vida, aumento no rendimento familiar e na escolaridade são citados como importantes fatores^6^. Além disso, a qualificação da oferta de serviços de saúde, a expansão da cobertura da Estratégia Saúde da Família e a transferência direta de renda são políticas exitosas com evidências de impacto positivo na redução dos óbitos infantis^7^.

No entanto, a segunda metade da década de 2010 no Brasil foi marcada por crise econômica, reformas em políticas sociais e econômicas, e limitação de gastos públicos, o que afetou especialmente programas e ações sanitárias e sociais com potencial para influenciar a mortalidade infantil. Estudos prévios estimaram os potenciais impactos dessas mudanças na saúde, indicando expressivos efeitos negativos em indicadores populacionais^8^. Também tem sido destacada a necessidade de avaliar se as tendências de redução das desigualdades passariam a ser revertidas^9^. A pandemia de covid-19 acentuou a preocupação quanto aos indicadores infantis, em particular pela forma desigual como atingiu o território brasileiro e pela alta carga da doença no país^10^.

O objetivo do presente estudo foi analisar a evolução das desigualdades socioeconômicas e regionais da mortalidade entre menores de cinco anos de idade no Brasil entre 2010 e 2022, identificando padrões e grupos de doenças com maior desigualdade e estimando excesso de óbitos ocorridos nos municípios com menor Índice de Desenvolvimento Humano (IDH).

## MÉTODOS

Foram analisados os dados de nascimentos e óbitos de todas as crianças menores de cinco anos de idade residentes no território brasileiro entre 2010 e 2022 por: faixa etária (menor de um ano de idade e entre um e quatro anos de idade), causa básica de óbito (segundo os capítulos da décima revisão da Classificação Internacional de Doenças – CID-10) e município e microrregião de residência.

O registro dos óbitos ocorridos no Brasil é compulsório e os dados são registrados em uma declaração de óbito que servirá para o registro civil do evento em cartórios oficiais. Todas as declarações de óbitos são coletadas pelas secretarias municipais de saúde e seus dados digitados na base nacional do Sistema de Informações sobre Mortalidade (SIM), gerenciado pelo Ministério da Saúde. De forma análoga, os registros de nascimento no Brasil também são obrigatórios e compõem o Sistema de Informações sobre Nascidos Vivos (Sinasc), que agrega os dados de todos os nascimentos ocorridos no território nacional.

Embora as coberturas do SIM e do Sinasc tenham aumentado significativamente desde os anos 2000, ainda persiste subregistro de estatísticas vitais no Brasil. Por isso o Instituto Brasileiro de Geografia e Estatística (IBGE) calcula as taxas de sub-registro e subnotificação de nascimentos e óbitos no Brasil e os disponibiliza de forma aberta^11^. Tais dados foram utilizados no presente estudo, incorporando-se as estimativas de nascimentos fornecidas pelo IBGE para cada município e as estimativas de subnotificação associadas aos dados do SIM, com o objetivo de corrigir os números de óbitos de crianças menores de cinco anos. Desde 2015, o IBGE disponibiliza estatísticas vitais corrigidas com base na técnica de captura-recaptura. Assim, para o período de 2010 a 2014, foram aplicadas as correções observadas no biênio 2015 e 2016 para cada município, levando em consideração a proximidade temporal e a necessidade de garantir a consistência dos ajustes com os dados mais recentes disponíveis e seguindo o mesmo método. Os valores globais de correção foram aplicados a cada capítulo de causas de óbitos. Adicionalmente, foi realizada análise de sensibilidade, replicando-se todos os cálculos a partir dos dados brutos do SIM e do Sinasc.

Os 5.565 municípios brasileiros emancipados em 2010 e, portanto, com dados censitários disponíveis para aquele ano foram agregados segundo os decis do Índice de Desenvolvimento Humano Municipal (IDH-M), de modo a compor grupos com quantidade de óbitos e de nascidos vivos que permitissem estabilidade nos indicadores. O IDH-M é uma adaptação do IDH da Organização das Nações Unidas (ONU) aplicada para medir o desenvolvimento humano em nível municipal no Brasil. Utilizou-se o índice calculado para 2010, ano inicial da análise. Tais dados são disponibilizados no Atlas de Desenvolvimento Humano^12^.

Inicialmente calcularam-se as taxas de mortalidade em cada um dos decis de IDH-M em cada ano analisado, permitindo a avaliação gráfica da série histórica em cada grupo. Em seguida, analisou-se para o ano final da série histórica, 2022, a taxa de mortalidade em cada decil de IDH-M segundo os 10 capítulos da CID-10 com maior ocorrência de mortes em cada faixa etária. O volume de óbitos nos demais capítulos se mostrou reduzido, limitando a possibilidade de análises segmentadas. Os capítulos com taxas calculadas foram: (1) Algumas doenças infecciosas e parasitárias, (2) Neoplasias, (3) Doenças do sangue e dos órgãos hematopoéticos, (4) Doenças endócrinas, nutricionais e metabólicas, (6) Doenças do sistema nervoso, (9) Doenças do aparelho circulatório, (10) Doenças do aparelho respiratório, (11) Doenças do aparelho digestivo, (16) Algumas afecções originadas no período perinatal, (17) Malformações congênitas, deformidades e anomalias cromossômicas, (18) Sintomas, sinais e achados anormais de exames clínicos e de laboratório, (20) Causas externas. Adicionalmente, calcularam-se as razões de mortalidade em cada capítulo entre os decis extremos de IDH-M.

Para analisar as desigualdades na ocorrência dos óbitos, estimaram-se para cada ano o *Slope Index of Inequality* (SII) e o *Relative Index of Inequality* (RII). O SII é considerado uma medida complexa de desigualdade que estima as diferenças nas taxas de mortalidade entre os extremos da distribuição socioeconômica considerando-se a composição de toda a amostra. Já o RII é uma medida relativa de desigualdade, e também considera tanto a distribuição da população entre diferentes grupos quanto a posição relativa de cada grupo.

Em seguida, foram calculados os excessos de óbitos em cada ano e decil de IDH-M assumindo-se para todos os decis os valores anuais de mortalidade observados no grupo de maior IDH-M. O cálculo do excesso de óbitos deu-se mediante a seguinte fórmula considerando-se idade(i) e ano(j):


$$\sum \left[{\mathrm{obtObs}}_{n}(i,j)-\left({\mathrm{txd}}_{10}(i,j)*{\mathrm{nv}}_{n}(i,j)\right)\right]$$

onde <0btObs_n_(i,j)> é o número de óbitos no decil n na idade i e ano j; < txd_10_(i,j)> é a taxa de mortalidade na idade i e ano j no decil de maior IDH-M; e < nv_n_(i,j)> é o número de nascidos vivos na idade i e ano j no decil n.

Por fim, para explorar a ocorrência de desigualdades regionais no território brasileiro, as taxas de mortalidade foram calculadas segundo as microrregiões de residência. O Brasil é dividido pelo IBGE em 558 microrregiões segundo proximidade geográfica, características socioeconômicas e integração funcional. A agregação geográfica permitiu cálculos mais estáveis nos valores calculados em comparação à análise por municípios, dada a existência no Brasil de muitos municípios com pequena população residente. Os mapas e as coordenadas geográficas foram obtidos junto ao IBGE, sendo a confecção dos mapas realizada no Python 3.4. As demais análises foram realizadas no Stata 15. Todos os dados de mortalidade e de nascimento são anonimizados e de domínio público, sendo disponibilizados pelo Ministério da Saúde^13^, dispensando-se, assim, apreciação do estudo em comitê de ética em pesquisa.

## RESULTADOS

Foram analisados os dados de 37.639.196 nascidos vivos, 483.900 óbitos entre crianças menores de um ano de idade e 79.811 óbitos entre crianças com um a quatro anos. O número de nascidos vivos foi de 2,91 milhões em 2010, atingindo seu valor máximo em 2015 (3,08 milhões), mas reduzindo nos anos seguintes até chegar a 2,57 milhões em 2022. A taxa de mortalidade infantil diminuiu de 13,0 por 1.000 nascidos vivos para 12,7 por 1.000 nascidos vivos entre 2010 e 2022 (Tabela). No entanto, observou-se que esse decréscimo ocorreu de forma sustentada apenas no primeiro quinquênio analisado. Em 2020 foi observado o valor mais baixo da série, mas com acréscimo nos anos seguintes e chegando em 2022 ao mesmo valor médio do quadriênio de 2014 a 2017. No caso da mortalidade entre crianças de um a quatro anos de idade, após redução até 2020, a taxa voltou a subir em 2021 e 2022 (Tabela). Ao final do período, passados 12 anos, o valor nominal foi idêntico ao observado em 2010.

**Tabela:** Óbitos, taxa de mortalidade, *Slope Index of Inequality* (SII), *Relative Index of Inequality* (RII) e razão e diferença absoluta entre decis extremos da taxa de mortalidade infantil e da taxa de mortalidade entre crianças de um a quatro anos de idade de acordo com os decis extremos do Índice de Desenvolvimento Humano Municipal. Brasil, 2010 a 2022.

Ano	Mortalidade infantil					
	Óbitos	Taxa	SII (IC95%)	RII (IC95%)	D1/D10	D1/D10
2010	40.947	13,0	-5,63 (-6,60 a -4,60)	0,69 (0,64 a 0,74)	1,48	5,90
2011	40.783	13,7	-5,61 (-6,78 a -4,43)	0,69 (0,63 a 0,74)	1,48	5,65
2012	40.226	13,6	-6,16 (-7,19 a -5,12)	0,66 (0,61 a 0,71)	1,55	6,42
2013	40.055	13,6	-6,68 (-7,45 a -5,91)	0,64 (0,60 a 0,67)	1,56	6,57
2014	39.503	13,0	-5,77 (-7,29 a -4,25)	0,66 (0,59 a 0,73)	1,56	6,39
2015	38.662	12,6	-5,14 (-6,89 a -3,39)	0,68 (0,60 a 0,77)	1,54	5,93
2016	37.240	12,9	-6,24 (-7,42 a -5,06)	0,64 (0,58 a 0,69)	1,58	6,46
2017	36.932	12,5	-5,64 (-7,01 a -4,26)	0,66 (0,59 a 0,72)	1,53	5,93
2018	36.562	12,3	-4,74 (-5,77 a -3,72)	0,70 (0,64 a 0,75)	1,47	5,06
2019	35.943	12,5	-4,98 (-6,79 a -3,17)	0,69 (0,60 a 0,78)	1,54	5,89
2020	32.046	11,6	-5,07 (-6,00 a -4,14)	0,67 (0,61 a 0,72)	1,50	5,12
2021	32.305	12,0	-5,05 (-7,73 a -2,38)	0,67 (0,54 a 0,81)	1,59	6,28
2022	32.695	12,7	-4,91 (-6,30 a -3,52)	0,70 (0,62 a 0,77)	1,49	5,44
	**Mortalidade entre crianças de um a quatro anos**					
2010	7.242	2,5	-2,42 (-3,24 a -1,60)	0,41 (0,29 a 0,54)	2,37	2,61
2011	6.877	2,3	-1,82 (-2,28 a -1,37)	0,49 (0,40 a 0,59)	2,07	1,92
2012	6.526	2,2	-1,77 (-2,53 a -1,01)	0,48 (0,34 a 0,63)	2,18	2,07
2013	6.537	2,2	-2,13 (-2,83 a -1,42)	0,42 (0,30 a 0,54)	2,35	2,29
2014	6.302	2,1	-1,80 (-2,87 a -0,74)	0,45 (0,25 a 0,66)	2,37	2,27
2015	5.783	1,0	-1,78 (-2,41 a -1,16)	0,42 (0,29 a 0,56)	2,29	1,90
2016	6.388	2,2	-1,93 (-3,10 a -0,77)	0,45 (0,24 a 0,66)	2,33	2,35
2017	6.056	2,0	-1,74 (-3,01 a -0,47)	0,46 (0,21 a 0,71)	2,30	2,18
2018	5.999	2,0	-1,38 (-1,98 a -0,79)	0,54 (0,39 a 0,68)	1,98	1,62
2019	5.934	2,1	-1,35 (-2,17 a -0,52)	0,55 (0,35 a 0,74)	1,97	1,70
2020	4.683	1,7	-1,48 (-2,07 a -0,90)	0,46 (0,32 a 0,60)	2,30	1,70
2021	5.104	1,9	-1,34 (-2,30 a -0,39)	0,52 (0,28 a 0,75)	2,10	1,74
2022	6.379	2,5	-1,71 (-2,59 a -0,82)	0,53 (0,36 a 0,70)	1,93	1,95

IC95%: intervalo de confiança de 95%.

As Figuras 1A e 1B mostram que houve claro gradiente de maiores taxas de mortalidade nos decis com menor IDH-M. Em todos os anos de 2010 a 2022, maiores taxas foram observadas nos municípios de menor IDH-M e taxas mais baixas foram identificadas nos municípios de maior IDH-M. Houve pequena oscilação nas posições dos municípios com índices intermediários. O SII reduziu de -5,63 para -4,91 na mortalidade infantil e de -2,42 para -1,71 na mortalidade de crianças entre um e quatro anos de idade (Tabela). Já o RII, na mortalidade infantil, oscilou discretamente para baixo nos primeiros quatro anos da série e passou a subir, sendo em 2022 discretamente superior ao valor inicial. Na mortalidade entre um e quatro anos, os valores de RII aumentaram de 0,41 para 0,53 (Tabela). Analisando-se a razão entre os dois decis extremos, a mortalidade infantil foi 48,0% e 49,0% maior no decil de menor IDH-M em 2010 e 2022, respectivamente. Na mortalidade entre crianças de um a quatro anos, os valores foram 137,0% e 93,0% maiores nos dois anos, respectivamente.

**Figura 1:** Série histórica da mortalidade infantil (A) e da mortalidade entre crianças de um a quatro anos de idade (B) nos municípios brasileiros segundo decis do Índice de Desenvolvimento Humano Municipal (IDH-M). Brasil, de 2010 a 2022.

Tais diferenças variaram de acordo com a causa dos óbitos. Agrupando-os segundo capítulos da CID-10, observou-se que a taxa de mortalidade infantil por doenças nutricionais, metabólicas e endócrinas foi 4,5 vezes maior no decil de menor IDH-M em comparação ao de maior desenvolvimento humano (Figura 2). Os valores foram também 3,0 vezes maiores no capítulo 18, que compreende causas mal definidas e desconhecidas de mortalidade e 2,5 vezes maiores no grupo de doenças infecciosas e parasitárias. Já entre as crianças de um a quatro anos, as maiores diferenças foram no grupo que contempla causas mal definidas e desconhecidas (3,5 vezes), doenças endócrinas, nutricionais e metabólicas (3,3 vezes), respiratórias (2,7 vezes), e infecciosas e parasitárias (2,7 vezes).

**Figura 2:** Taxa de mortalidade infantil (A) e entre crianças de um a quatro anos de idade (B) segundo capítulos da décima revisão da Classificação Internacional de Doenças de acordo com decis do Índice de Desenvolvimento Humano Municipal (IDH-M), e razão entre os decis de menos e de maior IDH-M. Brasil, 2022.

Tais desigualdades resultaram em expressiva quantidade de óbitos infantis em excesso no Brasil. Caso as taxas de mortalidade observadas no grupo de maior IDH-M fossem aplicadas aos demais decis, teriam sido evitadas 76.832 mortes de crianças menores de cinco anos de idade entre 2010 e 2022 (Figura 3A). O excesso de óbitos infantis foi maior entre 2010 e 2013, reduzindo sobretudo a partir de 2017 (acompanhando a redução de nascidos vivos), mas se mantendo sempre acima de 4.500 por ano. Os valores de excesso de óbitos também foram maiores no decil de menor IDH-M, atingindo quase 16.164 mortes em excesso apenas nesses municípios entre 2010 e 2022 (Figura 3B).

**Figura 3:** Número de óbitos em excesso comparando-se as taxas de mortalidade infantil e entre crianças de um a quatro anos dos decis de menor Índice de Desenvolvimento Humano Municipal (IDH-M) em comparação às taxas observadas no decil de maior IDH-M segundo ano do óbito (A) e segundo decil socioeconômico de todo o período (B). Brasil, de 2010 a 2022.

A análise espacial dos dois desfechos analisados também mostrou a existência de profundas e constantes desigualdades regionais no Brasil. Observou-se que as regiões Norte e Nordeste concentraram a maior parte das microrregiões com taxas mais altas de mortalidade (Figura 4). Em 2022, 42,2% e 28,2% das microrregiões do Norte e do Nordeste estavam entre as 100 com maiores taxas de mortalidade infantil, valores que chegaram a apenas 5,0%, 3,2% e 17,3% nas regiões Sudeste, Sul e Centro-Oeste, respectivamente. Além disso, somente 7,8% e 9,6% das microrregiões do Norte e do Nordeste estavam entre as 100 com menores taxas de mortalidade infantil, enquanto nas regiões Centro-Oeste, Sul e Sudeste os valores foram 17,3%, 31,9% e 23,8%, respectivamente.

**Figura 4:** Taxa de mortalidade infantil em 2010 (A) e 2022 (B) e entre crianças de um a quatro anos de idade em 2010 (C) e 2022 (D) segundo microrregião de residência da criança. Brasil, 2010 e 2022.

Na análise de sensibilidade, observaram-se variações em todas as medidas calculadas quando empregados os dados não corrigidos do SIM e do Sinasc.

No entanto, as diferenças foram de baixa magnitude e não modificaram a direção das associações, conforme pode ser observado no material suplementar^a^.

## DISCUSSÃO

O presente estudo encontrou cinco importantes resultados. Primeiro, houve discreta redução na mortalidade de menores de cinco anos de idade no período analisado, com pequena oscilação entre 2016 e 2019 e piora em 2022. Segundo, as desigualdades na mortalidade persistiram ao longo de todo o período, com apenas redução discreta ou estabilização. Terceiro, as desigualdades foram mais agudas nos óbitos por doenças nutricionais, metabólicas e endócrinas, doenças infecciosas e parasitárias, respiratórias e no grupo de causas mal definidas. Quarto, se os municípios tivessem as mesmas taxas de mortalidade observadas no decil de maior IDH-M, quase 77 mil óbitos de menores de cinco anos poderiam ter sido evitados no Brasil entre 2010 e 2022. Por fim, profundas desigualdades regionais na mortalidade perpassaram os anos, com piores indicadores nas regiões Norte e Nordeste e com pouca oscilação.

A redução da mortalidade observada em alguns momentos da série histórica reflete melhorias nas condições de vida e no acesso a serviços de saúde. Estudos anteriores já mostraram que melhorias econômicas, sanitárias e sociais associadas a intervenções na atenção primária à saúde (APS) e nos programas de transferência de renda contribuíram significativamente para a redução da mortalidade entre menores de cinco anos de idade no Brasil^7,14^. No entanto, a relativa estabilidade entre 2016 e 2019 e o aumento em 2022 indicam que esses avanços não são conquistas definitivas. A revisão da Política Nacional da Atenção Básica (PNAB) em 2017 foi citada como ameaça ao fortalecimento da atenção primária no Brasil ao relativizar a cobertura universal^15^. Freire et al.^16^ observaram, nos dois anos seguintes à aprovação da nova PNAB, o crescimento dos municípios que reduziram o número de agentes comunitários de saúde atuando no Sistema Único de Saúde (SUS). Outras medidas, como o desestímulo a equipes multidisciplinares de apoio à APS e as mudanças no financiamento e avaliação da atenção primária, também foram alvo de críticas no meio científico^17^.

Na base desse cenário desafiador à saúde esteve a Emenda Constitucional 95, que estabeleceu, a partir de 2017, o congelamento dos investimentos públicos no Brasil por 20 anos^18^. A proposta foi substituída a partir de 2023; no entanto, restringiu o financiamento público em saúde durante período importante de crise econômica^18^. Estudos prévios mostraram impactos significativos de medidas de desfinanciamento de ações no setor saúde^8,19^. Além disso, comparativamente a 2013, o financiamento federal em 2019 foi menor nas áreas de habitação, educação, saneamento, trabalho, urbanismo, desenvolvimento rural e cultura^20^.

A maior desigualdade nas doenças nutricionais, metabólicas e endócrinas, além das infecciosas e parasitárias e das doenças respiratórias, pode ser explicada por distintas exposições que moradores de municípios com diferentes índices de desenvolvimento humano apresentam. No Brasil, regiões mais desprivilegiadas apresentam maior insegurança alimentar e nutricional^21^, menor acesso à infraestrutura, à moradia adequada e a saneamento básico^22^ e maiores dificuldades de acesso aos serviços de saúde^23^. Já a maior taxa de mortalidade pelas causas constantes do capítulo 18 (sintomas, sinais e achados anormais de exames clínicos e de laboratório) em municípios com menor IDH-M pode estar relacionada à maior limitação no acesso a diagnósticos precisos e recursos em saúde adequados, além de fragilidades nos sistemas de vigilância e investigação de óbitos.

Tais contextos estruturais têm potencial de impactar mais severamente as populações em situação de vulnerabilidade e localidades com menos recursos e serviços públicos estruturados. Observou-se que, apesar de algumas melhorias, as desigualdades permanecem significativas e persistentes. Estudo de abrangência nacional^24^ já mostrou que entre 2013 e 2019 houve aumento na prevalência de oito doenças crônicas e que as desigualdades presentes em sua distribuição não se modificaram no período. Já o estudo de Sousa et al.^25^ identificou que já nos primeiros anos da crise econômica e política brasileira (2015 a 2017) houve deterioração da sensação de bem-estar no país, tendo ocorrido redução mais acentuada entre pessoas com menor escolaridade e com menor suporte social.

Os acréscimos nas taxas de mortalidade observadas no presente estudo em 2021 e 2022 podem estar, ao menos parcialmente, associadas a efeitos da pandemia pela covid-19. Shapira et al.^26^ reportaram aumento de 6,8% no número total de mortes infantis esperadas em 2020 no somatório de 83 países, fato que estaria associado a choques negativos de renda por conta da pandemia. Já Ahmed et al.^27^ argumentaram que o declínio na utilização dos serviços essenciais de saúde pode explicar o acréscimo de óbitos infantis nos dois primeiros anos da pandemia. Em 2020, o Brasil ainda apresentou queda na mortalidade entre menores de cinco anos. É possível que, no primeiro ano da pandemia, medidas de mitigação tenham contribuído para reduzir a exposição a patógenos causadores de doenças na idade pediátrica. Já nos anos seguintes, a mortalidade na infância pode ter sido impactada pela pletora dos serviços de saúde e acesso retardado a eles, pela retomada das atividades sociais em um contexto de baixa organização coletiva e governamental de enfrentamento à pandemia e pelos efeitos da crise econômica.

Outro achado importante é que a quantidade de óbitos em excesso mostrou-se bastante expressiva. Esses números revelam a profunda desigualdade ainda existente no Brasil e que permeia diversas dimensões da vida. Apesar de todos os brasileiros serem legalmente protegidos pelos mesmos direitos fundamentais e de haver um sistema de saúde público e universal, há significativa sobremortalidade nos municípios com menor IDH-M. Isso mostra que os conhecimentos produzidos e as tecnologias disponíveis não são disponibilizados e acessados com equidade. Adicionalmente, diante de mais de uma década com poucos avanços no enfrentamento às desigualdades, é preciso avaliar na gestão pública se está ocorrendo o monitoramento das desigualdades e a implementação de ações equânimes baseadas nas melhores evidências e de acordo com a leitura dos dados em tempo real. Padrão semelhante foi observado na Inglaterra, com 9.294 óbitos de menores de ano em excesso entre 2009 e 2020^28^. Entretanto, ao se analisar os óbitos em excesso em todas as idades, diferentemente do observado entre crianças no Brasil, a quantidade na Inglaterra aumentou ao longo do tempo, em especial a partir de 2015 e com pico em 2020. Ao interpretar o caso brasileiro, destaca-se a redução importante no número de nascidos vivos a partir de 2019.

As desigualdades espaciais sugerem que há insuficiência nas políticas públicas voltadas para a equidade regional. A concentração de microrregiões com altas taxas de mortalidade infantil no Norte e Nordeste expressam desafios, como dificuldade de acesso aos serviços de saúde de qualidade, menor infraestrutura no setor saúde e condições socioeconômicas mais desfavoráveis^19,29-30^. Esses achados indicam a necessidade de políticas públicas efetivas focadas em reduzir as disparidades regionais.

Limitações deste estudo incluem a possível subnotificação residual de óbitos e a variação na qualidade dos dados entre diferentes regiões. Ao empregar a correção dos dados procurou-se enfrentar essa limitação, mas variações de qualidade podem permanecer, ainda que com menor expressão. Além disso, a análise de sensibilidade com dados brutos mostrou ajustes apenas modestos nas medidas calculadas. Uma segunda limitação é a agregação das análises em decis de IDH-M e microrregiões, o que não capturaria a heterogeneidade que pode existir dentro desses grupos. No entanto, tal medida é importante para conferir estabilidade aos indicadores calculados. Um terceiro aspecto a ser considerado é que as mudanças nas políticas e na economia no período de estudo podem ter efeitos complexos e de longo prazo sobre a mortalidade infantil e que não foram completamente capturados. Por fim, índices agregados, como o IDH-M, podem não capturar adequadamente as complexidades de populações com alta diversidade étnico-racial, como a brasileira. No entanto, o índice reflete condições socioeconômicas amplamente conhecidas por impactarem a saúde infantil de forma robusta e oferece um instrumento uniforme que facilita a comparação entre diferentes municípios.

Os resultados têm importantes implicações para políticas públicas. Intervenções focadas em reduzir as desigualdades regionais e socioeconômicas são essenciais para melhorar a saúde infantil no Brasil. Políticas econômicas, sociais e de saúde precisam ser implementadas para acelerar a melhoria das condições gerais de vida e do ambiente de moradia e qualificar o acesso aos serviços de saúde. Ainda, a pandemia de covid-19 trouxe novos desafios, acentuando desigualdades preexistentes e destacando a necessidade de resiliência nos sistemas de saúde. Além disso, é preciso ampla discussão entre gestores de todos os níveis sobre como monitorar e reduzir desigualdades em saúde.

## Footnotes

a Disponível no repositório institucional da Universidade Federal de Santa Catarina em: https://repositorio.ufsc.br/handle/123456789/260749#:~:text=material_suplementar.pdf.
